# Investigation of the feasibility of NRAV as a biomarker for hepatocellular carcinoma

**DOI:** 10.32604/or.2023.043575

**Published:** 2024-03-20

**Authors:** JUN LIU, WENLI LI, RUYUE LU, JIAQING XU, CHUNHUI JIANG, JUNLIN DUAN, LINGZHI ZHANG, GUANFU WANG, JIAXI CHEN

**Affiliations:** 1 Department of Clinical Laboratory, Taizhou Hospital of Zhejiang Province Affiliated to Wenzhou Medical University, Linhai, China; 2 Key Laboratory of System Medicine and Precision Diagnosis and Treatment of Taizhou, Taizhou, China; 3 Department of Clinical Laboratory, Dongguan Hospital of Traditional Chinese Medicine, Dongguan, China; 4 School of Basic Medical Sciences Forensic Medicine, Hangzhou Medical College, Hangzhou, China

**Keywords:** NRAV, Hepatocellular carcinoma, Prognosis, Biomarker, Pan-cancer

## Abstract

The long non-coding RNA, Negative Regulator of Antiviral Response (NRAV) has been identified as a participant in both respiratory virus replication and immune checkpoints, however, its involvement in pan-cancer immune regulation and prognosis, particularly those of hepatocellular carcinoma (HCC), remains unclear. To address this knowledge gap, we analyzed expression profiles obtained from The Cancer Genome Atlas (TCGA) database, comparing normal and malignant tumor tissues. We found that NRAV expression is significantly upregulated in tumor tissues compared to adjacent nontumor tissues. Kaplan-Meier (K-M) analysis revealed the prognostic power of NRAV, wherein overexpression was significantly linked to reduced overall survival in a diverse range of tumor patients. Furthermore, noteworthy associations were observed between NRAV, immune checkpoints, immune cell infiltration, genes related to autophagy, epithelial-mesenchymal transition (EMT), pyroptosis, tumor mutational burden (TMB), and microsatellite instability (MSI) across different cancer types, including HCC. Moreover, NRAV upregulation expression was associated with multiple pathological stages by clinical observations. Furthermore, our investigation revealed a substantial elevation in the expression of NRAV in both HCC tumor tissues and cells compared to normal tissues and cells. The inhibition of NRAV resulted in the inhibition of cell proliferation, migration, and invasion in HCC cells, while also influencing the expression of CD274 (PD-L1) and CD44, along with various biomarkers associated with EMT, autophagy, and pyroptosis. The aforementioned results propose NRAV as a promising prognostic biomarker for HCC.

## Introduction

Cancer poses a significant global health burden, characterized by escalating rates of morbidity and mortality. To date, cancer treatment remains challenging [1]. With the constant emergence of novel concepts and technologies in cancer interventions, the need to establish accurate approaches for the diagnosis, prevention, and treatment of cancers has become progressively more crucial [2]. In the last decade, the exploration of easily identifiable molecular markers and essential functional attributes of pan-cancer profiles, with the objective of better understanding the underlying molecular mechanisms of cancer, has constituted a primary area of concentration within the field of cancer research [3–7].

The expression of genes is controlled by numerous gene regulatory factors, such as noncoding RNAs (ncRNAs) and transcription factors [8]. Studies have shown that long non-coding RNAs (lncRNAs), with a length of approximately 200 nucleotides, have been demonstrated to exert a pivotal influence on various biological processes, while also exhibiting significant potential in the realms of complex disease diagnosis, prevention, and treatment. Specifically, lncRNAs are dysregulated in various types of cancer and may function through gene networks that contribute to cancer development [9]. Although the significance of lncRNAs in diverse biological processes and complex diseases is well-established, limited research has investigated the direct correlation between lncRNAs and pan-cancer.

One lncRNA, Negative Regulator of Antiviral Response (NRAV) is involved in the host antiviral responses and regulation of virus replication [10,11]. Some studies have shown that NRAV play a role in immune checkpoints in hepatocellular carcinoma (HCC), while other studies have identified ferroptosis as a possible mechanism [6,12]. Interestingly, NRAV’s initial discovery was associated with its role in modulating innate immune responses against viral infections [13]. However, there remains a lack of comprehensive understanding regarding the precise function of NRAV in different tumor types. Thus, the objective of this study was to systematically investigate additional potential roles of NRAV in various human tumors by analyzing lncRNA expression profiles obtained from TCGA. Furthermore, we aimed to quantitatively assess the influence of aberrant NRAV expression on the unfavorable prognosis of cancer patients, utilizing follow-up clinical data. NRAV expression was also examined in relation to TMB, MSI, and pathway-related molecules. By incorporating the analysis of NRAV’s expression and prognostic significance with an *in vitro* functionality investigation, we can deduce potential mechanisms elucidating the interaction between NRAV and tumor immunity, thereby highlighting NRAV’s potential role in HCC and other malignancies.

## Materials and Methods

### Data collection

All analyses were performed using gene expression and lncRNA datasets obtained from the TCGA Research Network (http://cancergenome.nih.gov/). We acquired data on raw read count and fragments per kilobase of transcript per million mapped reads-based (FPKM) data were acquired for 33 cancer types and transformed the data to Transcripts per million (TPM) formats. Additionally, we collected clinical data on those patients with cancer, including stage, grade, survival status, and clinical follow-up survival information, were obtained. In the section pertaining to Clinical Relevance Analysis, patients whose pathology staging was incomplete were excluded. Furthermore, a cohort of 16 patients diagnosed with hepatocellular carcinoma (HCC) at Enze Hospital was included in our study. These patients were newly diagnosed at Enze Hospital and had not received any targeted or immunotherapy interventions, with surgical resection being the sole treatment modality. Following the ethical endorsement of the Enze Hospital Ethics Committee and the acquisition of informed consent from the patients, we conducted this study.

### Differential analysis and prognostic analysis

The entire dataset underwent filtration using “RMA” packs to eliminate duplicated and missing results, followed by log2 (TPM +1) conversion. Two-tailed Wilcoxon rank-sum tests were employed to assess the significant differences in NRAV expression between normal and tumor tissues across various tumor types. NRAV expression values were then categorized as high (expression value > median) or low (expression value < median) using the “SurvMiner” and “Survival” packages. Kaplan-Meier curves were generated to compare predicted high and low groups and assess differences in survival between the high and low expression groups in various types of tumors. The findings were verified using the log-rank test.

### Correlation analysis

Tumor Immune Estimation Resource (TIMER, https://cistrome.shinyapps.io/timer/) was used to evaluate the correlation between NRAV expression and immune cell infiltration. The TIMER database offers information on the presence of six distinct types of immunocytes infiltrating in 33 tumor types from TCGA. The GSVA package performed a single sample Gene Set Enrichment Analysis (ssGSEA) to explore the relationship between NRAV expression and immune cell infiltration. To better understand the roles of TMB and MSI in anti-cancer immunotherapy, specifically associations with immune checkpoints, epithelial-mesenchymal transition (EMT), autophagy and pyroptosis in malignant proliferation and metastasis, we looked for correlations between NRAV expression and key pathway-related genes.

### Expression analysis of NRAV at the single-cell level in HCC

Tumor Immune Single-cell Hub 2 (TISCH2) is a valuable single-cell database (http://tisch.comp-genomics.org/home/) that integrates gene expression data with tumor immune cell infiltration information. In our study, we leveraged TISCH2 to analyze the expression of NRAV in HCC at the single-cell level.

### Clinical prognosis and NRAV expression in HCC patients

The Database of LIHC expression atlas (HCCDB), specifically GSE36376, GSE54236, and GSE76427 were used to evaluate the expression of NRAV in HCC. We than examined the relationship between NRAV expression and various clinical factors, including clinical stage, tumor stage, histologic grade, and vascular invasion. Finally, the prognostic value was analyzed by constructing Receiver Operating characteristic curve (ROC) methods.

### Cell culture

The HCC cell lines, specifically Huh7 and HCCLM3 were cultivated in the laboratory. The precise sequence of the human target gene NRAV short hairpin RNA (shRNA) was procured from GenScript (https://www.genscript.com/) and is presented below: NRAV shRNA-1: gGGTCTTGTGTTAACTCTTAttcaagagaTAAGAGTTAACACAA

GACCttttt, NRAV shRNA-2: gGCAAAGTGGTCTTGTGTTAttcaagagaTAACACAA

GACCACTTTGCttttt, NRAV shRNA-3: gGGATGGATAGTTCAGAGTAttcaagagaT

ACTCTGAACTATCCATCCttttt. After that, Lipo3000 and plasmids were transfected into cells.

The reverse transcription PCR (RT-PCR) and Western blot analysis.

A kit from Beyotime (https://www.beyotime.com) was used for RT-PCR, while an ExicyclerTM 96 quantitative fluorescence analyzer was employed for fluorescence signals detection. All results were all normalized to GAPDH after processing with the 2^−ΔΔ^ Ct method. The Primer sequences used include: NRAVf: TCTCAACCTTGGCACTA, NRAVr: CAGAAGACATTCGGCAC; β-actinf: GGCACCCAGCACAATGAA, and β-actinr: TAGAAGCATTTGCGGTGG. The following antibodies were used in the western blot analysis: N-cadherin antibody (wanleibio, WL01047, 1:500, Shenyang, China), E-cadherin antibody (wanleibio, WL01482, 1:400, Shenyang, China) Vimentin antibody (wanleibio, WL01960, 1:500, Shenyang, China) Cleaved caspase-1 antibody (wanleibio, WL02996a, 1:1000, Shenyang, China) NLRP3 antibody (wanleibio, WL02635, 1:500, Shenyang, China) IL1β antibody (wanleibio, WL00891, 1:500, Shenyang, China) IL-18 antibody (wanleibio, WL01127, 1:500, Shenyang, China) CD274 antibody (wanleibio, WL02778, 1:1000, Shenyang, China) CD44 antibody (wanleibio, WL03531, 1:1000, Shenyang, China) ATG7 antibody (wanleibio, WL02793, 1:500, Shenyang, China) ATG12 antibody (wanleibio, WL03144, 1:1000, Shenyang, China) ATG16L1 antibody (wanleibio, WL02404, 1:500, Shenyang, China) LC3-II/I antibody (wanleibio, WL01506, 1:500, Shenyang, China) Sheep anti-rabbit IgG-HRP (wanleibio, WL023, 1:5000, Shenyang, China), and β-actin (wanleibio, WL01845, Shenyang, China).

### Cell counting kit-8 (CCK8) assays

Cell assays were conducted using a CCK8 detection kit (WLA074, Shenyang, China) in adherence to the manufacturer’s instructions. HCC cells at approximately 90% confluence were rinsed twice with PBS and treated with 0.25% trypsin for digestion. Subsequently, exponentially growing cell lines were seeded into 96-well plates at a density of 4 * 103 cells per well. With a cell counting kit-8 (WLA074, Shenyang, China), cell viability was assessed after 24, 48, 72, and 96 h.

### Wound healing asaay

Huh7 and HCCLM3 cells were seeded into six-well plates at a density of 1000 cells per well using Huh7 and HCCLM3 cell lines. Following 48 h of transfection, a scratch wound was created on confluent cells using a 200 mL pipette tip. The cell debris was eliminated through two washes with a serum-free medium. The gap size was measured and the wound areas were photographed at 0, 24, and 48 h. Microscopic examination at 100× magnification revealed observable morphological alterations.

### Migration/invasion tests

The Boyden chambers manufactured by BD Biosciences were employed to conduct migration and invasion experiments, while a transwell plate with an 8 μm aperture (Corning, Costar) was employed to measure the migration and invasion of cells. This plate is equipped with an 8 μm aperture sourced from Corning Costar in the United States. For each experimental group, a cell suspension of 200 μL was added to the Transwell plates as an inoculum. The upper chamber was filled with serum-free medium, while the lower chamber contained culture medium. Following a 24-h incubation period, a fixation solution consisting of 4% paraformaldehyde (PFA) was applied for 20 min at room temperature to immobilize the cells. Afterwards, 0.5% crystal violet was added to enhance visibility.

## Results

### The NRAV expression in pan-cancer

**Figure 1:**
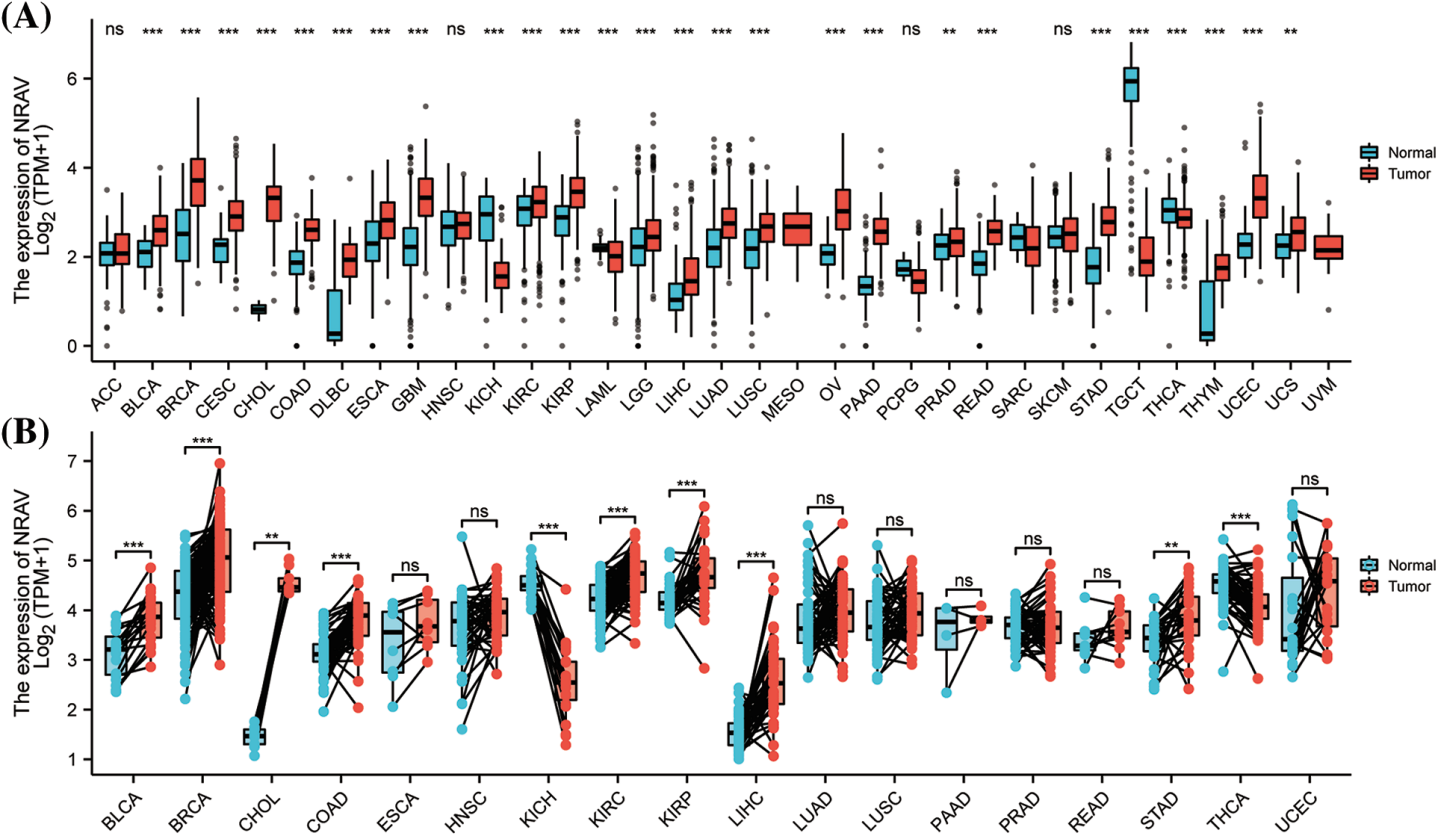
A comparison of NRAV expression levels. (A) The pan-cancer mRNA expression of NRAV. (B) NRAV expression differences were compared between tumor and adjacent tissues using Mann-Whitney U test. ns, *p* ≥ 0.05; **p < 0.01; ***p < 0.001.

A comprehensive review of TCGA mRNA data allowed for the study of NRAV expression patterns in 33 TCGA cancer types. The findings indicated a significant upregulation of NRAV expression in the majority of tumors, particularly in BLCA, bladder urothelial carcinoma; BRCA, breast invasive carcinoma; CESC, cervical and endocervical cancers; CHOL, cholangiocarcinoma; COAD, colon adenocarcinoma; DLBC, lymphoid neoplasm diffuse large B-cell lymphoma; ESCA, esophageal carcinoma; GBM, glioblastoma multiforme; KICH, kidney chromophobe; KIRC, kidney renal clear cell carcinoma; KIRP, kidney renal papillary cell carcinoma; LAML, acute myeloid leukemia; LGG, brain lower grade glioma; LIHC, liver hepatocellular carcinoma; LUAD, lung adenocarcinoma; LUSC, lung squamous cell carcinoma; OV, ovarian serous cystadenocarcinoma; PAAD, pancreatic adenocarcinoma; PRAD, prostate adenocarcinoma; READ, rectum adenocarcinoma; STAD, stomach adenocarcinoma; STES, stomach and esophageal carcinoma; TGCT, testicular germ cell tumors; THCA, thyroid carcinoma; THYM, thymoma; UCEC, uterine *corpus* endometrial carcinoma; and UCS, uterine carcinosarcoma. However, we found that NRAV expression was decreased in SARC, sarcoma (Fig. 1A), and there were no significant differences in NRAV expression were observed in ACC, adrenocortical carcinoma; HNSC, head and neck squamous cell carcinoma; PCPG, pheochromocytoma and paraganglioma; or SKCM, skin cutaneous melanoma. Paired comparison analyses were performed and results showed overexpression of NRAV in tumor tissues in BLCA, BRCA, CHOL, COAD, KIRC, KIRP, LIHC, STAD, and THCA (Fig. 1B).

### Survival analysis

**Figure 2:**
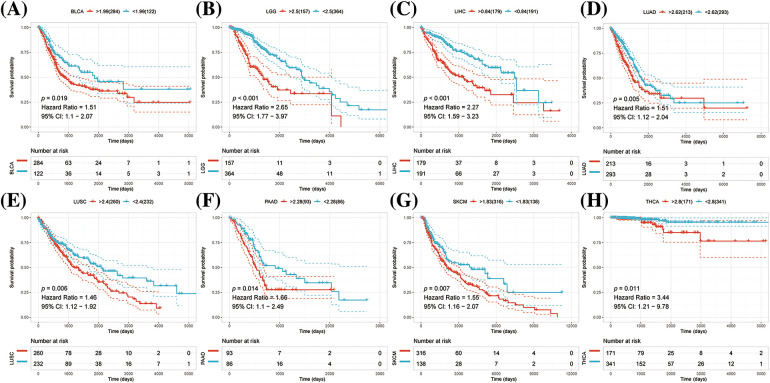
Pan-cancer survival analysis of NRAV. Based on Kaplan-Meier analysis, we determined whether NRAV expression affected the overall survival rate (OS) of LGG (A), LIHC (B), LUAD (C), LUSC (D), PAAD (E), PRAD (F), MESO (G) and THCA (H).

K-M survival analysis was performed to examine any possible association between abnormal expression of NRAV and overall survival (OS) and disease-specific survival (DSS). The results suggested that patients with high levels of NRAV expression had shorter OS compared to those in the low NRAV expression group in LGG, LIHC, LUAD, LUSC, PAAD, PRAD, MESO and THCA (Figs. 2A–2H).

### Analysis NRAV expression and tumor microenvironment in gene sets

Cancer microenvironment consists mainly of immune cells surrounding tumor cells, apart from the tumor cells themselves. Immune cell infiltration influences survival and tumor metastasis. To gain a deeper comprehension of the relationship between abnormal NRAV expression and immune cell infiltration, we conducted an analysis on the immune cell scores of six cancer types using the online TIMER database. Our objective was to ascertain the correlation between NRAV expression and levels of immune cell infiltration. The findings revealed a significant positive correlation between NRAV and B cells, CD4+ T cells, CD8+ T cells, Neutrophil, Macrophage, and DC cells in KIRC, LGG, PRAD, LIHC, USC, GBMLGG, KICH, and DLBC (Suppl. Fig. S1A). We then investigated the relationship between NRAV and immune checkpoint genes, and found that expression was positively correlated with several immune checkpoint inhibitors, especially CD274 and CD276 in LGG and LIHC, however, a significant negative correlation was found between NRAV and many immune checkpoints in various cancers, including BRCA, CESC, ESCA, SKCM, and THCA (Suppl. Fig. S1B). The NRAV and autophagy-related genes were positively correlated in BRCA, DLBC, GBM, PAAD, PRAD, TGCT, and especially in LIHC (Suppl. Fig. S1C).

Metastasis is widely recognized as the primary contributor to cancer-related mortality, with EMT playing a pivotal role in its advancement. Consequently, we investigated the potential association between NRAV and tumor metastasis by examining the expression correlation with EMT-related molecules. NRAV and EMT-related molecules were statistically significantly correlated in KIRC, LGG, LIHC, and TGCT (Suppl. Fig. S2A). In addition, NRAV expression was correlated with RRAS, MTHFD2, DAB2 and PPIC in a variety of tumors, but negatively correlated with MMP2, CCL2, IL1 and GAS1 expression in a wide variety of tumors showed a significant negative correlation (Suppl. Fig. S2A). Pyroptosis, a novel form of programmed inflammatory cell death characterized by necrotic morphologies such as plasma membrane rupture, plays a crucial role in tumor development, therefore, we investigated the association between NRAV expression and pyroptosis-related molecules. The results showed that NRAV was positively correlated with pyroptosis-related molecules across several cancer types, including COAD, GBM, KICH, LGG, LIHC, PAAD, and TGCT. Furthermore, NRAV exhibited a significant positive correlation with IL8, CHMP2A, CHMP4B, GSDMD, CASP1/3, and CHMP6 across various tumor types (Suppl. Fig. S2B). Genetic components, such as tumor mutation burden (TMB) and microsatellite instability (MSI) are associated with better responses to checkpoint inhibitors and increased T-cell infiltration in cancers, therefore, we examined whether NRAV expression was associated with MSI or TMB across 33 TCGA cancer types. In THYM, SKCM, PAAD, KIRC, BRCA, and BLCA, NRAV expression was positively correlated with TMB, but negatively correlated with TMB in DLBC, COAD, BRCA, THCA, LIHC, KIRC and HNSC (Suppl. Figs. S2C and S2D).

### Co-expression correlation and functional enrichment analysis

Using a single-sample gene set enrichment analysis (ssGSEA) algorithm, we detected 24 types of immune cells infiltration from 374 TCGA-LIHC samples. Results revealed significant positive correlations between NRAV and Th2 cells, TFH, macrophages, NK CD56 bright cells, and T helper cells. While Cytotoxic cells, Th17 cells, DC, pDC, Tgd, Treg, Neutrophils and CD8 T cells presented significant negative correlation with NRAV expression in HCC (Suppl. Fig. S3A). Further, gene expression correlation analysis identified 74 related genes based on the following criteria: cor-pearson > 0.6 and *p*-value < 0.001. Finally, a heatmap was constructed to visualize the correlation between NRAV expression and the top 20 co-expression genes (Suppl. Fig. S3B).

To further explore the the regulatory mechanisms of NRAV co-expression genes, we attempted to identify possible pathways using Gene Ontology (GO) and Kyoto Encyclopedia of Genes and Genomes (KEGG) enrichment analysis. The cellular components enriched in the NRAV co-expression genes included the ciliary part, spindle, and kinetochore (Suppl. Fig. S3C). The GO enrichment analysis revealed that these molecules were primarily associated with adhesion molecule binding, ATPase activity, tubulin binding, cadherin binding, microtubule binding, transferase activity, p53 binding, and histone acetyltransferase activity. Furthermore, the KEGG analysis demonstrated that the NRAV co-expression genes were predominantly involved in RNA transport, cell cycle, ribosome biogenesis in eukaryotes, and vasopressin-regulated water reabsorption, as shown in Suppl. Fig. S3D.

### Clinical correlation analysis of NRAV in HCC

**Figure 3:**
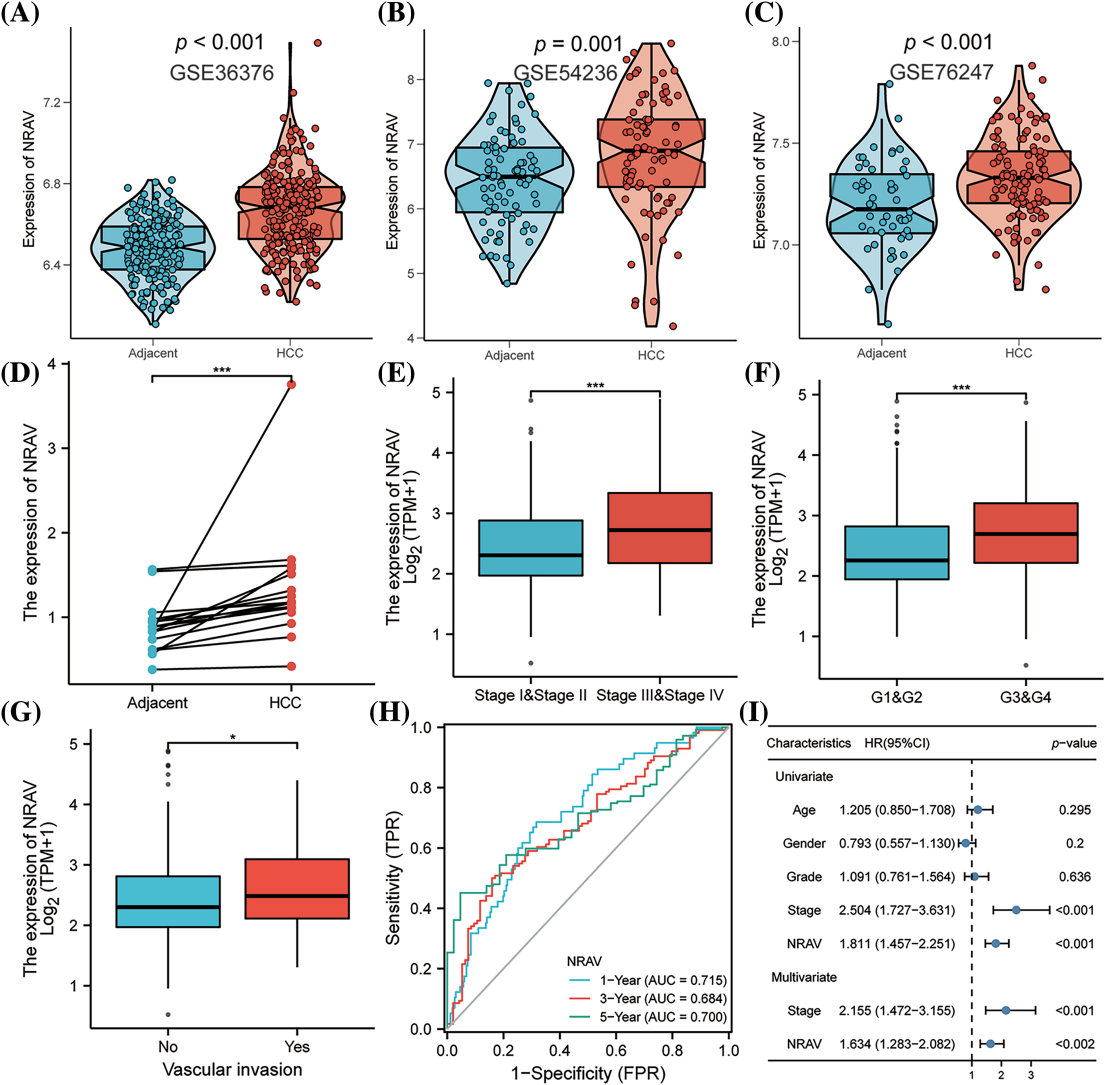
Correlations between clinico-pathological parameters and NRAV expressions. (A–D) Validation of AHSA1 expressions in HCC. Variation analysis of NRAV expression in pathologic T stage (E), histologic grade (F) and vascular invasion (G). ROC analysis (H) and COX analysis (I) were used to analyze the prognostic significance of NRAV in HCC. *p < 0.05, ***p < 0.001.

These findings collectively indicate that NRAV plays a significant role in the development of HCC. Additionally, we also found that the clinical stage of HCC was associated with NRAV expression. Analysis of gene expression data from GSE36376, GSE54236, and GSE76247 revealed a substantial upregulation of NRAV in HCC tissues compared to that in normal liver tissues (Figs. 3A–3C). To further validate these findings, the expression of NRAV in a local cohort was assessed using RT-PCR, which confirmed the upregulation of NRAV in tumor tissues (Fig. 3D). In addition, we also found that upregulated NRAV expression was also related to the malignant pathological progression, specifically a higher pathologic T stage (Fig. 3E), and higher histologic grade (Fig. 3F), and greater vascular invasion (Fig. 3G). We then analyzed the prognostic power of NRAV in patients with HCC using the ROC method. The results indicated a favorable performance in prognostic evaluation, the AUC values for 1, 3, and 5 years were 0.715, 0.684, and 0.700, respectively (Fig. 3H). Finally, Cox regression analyses demonstrated that NRAV independently served as a prognostic factor for HCC (Fig. 3I). Furthermore, we conducted additional investigation into the single-cell expression level of NRVA in hepatocellular carcinoma utilizing TISCH2. The findings revealed a higher abundance of NRVA expression in both fibroblasts and malignant cells (Suppl. Figs. S4A–S4C).

### The role of NRAV in cell invasion and migration of HCC

**Figure 4:**
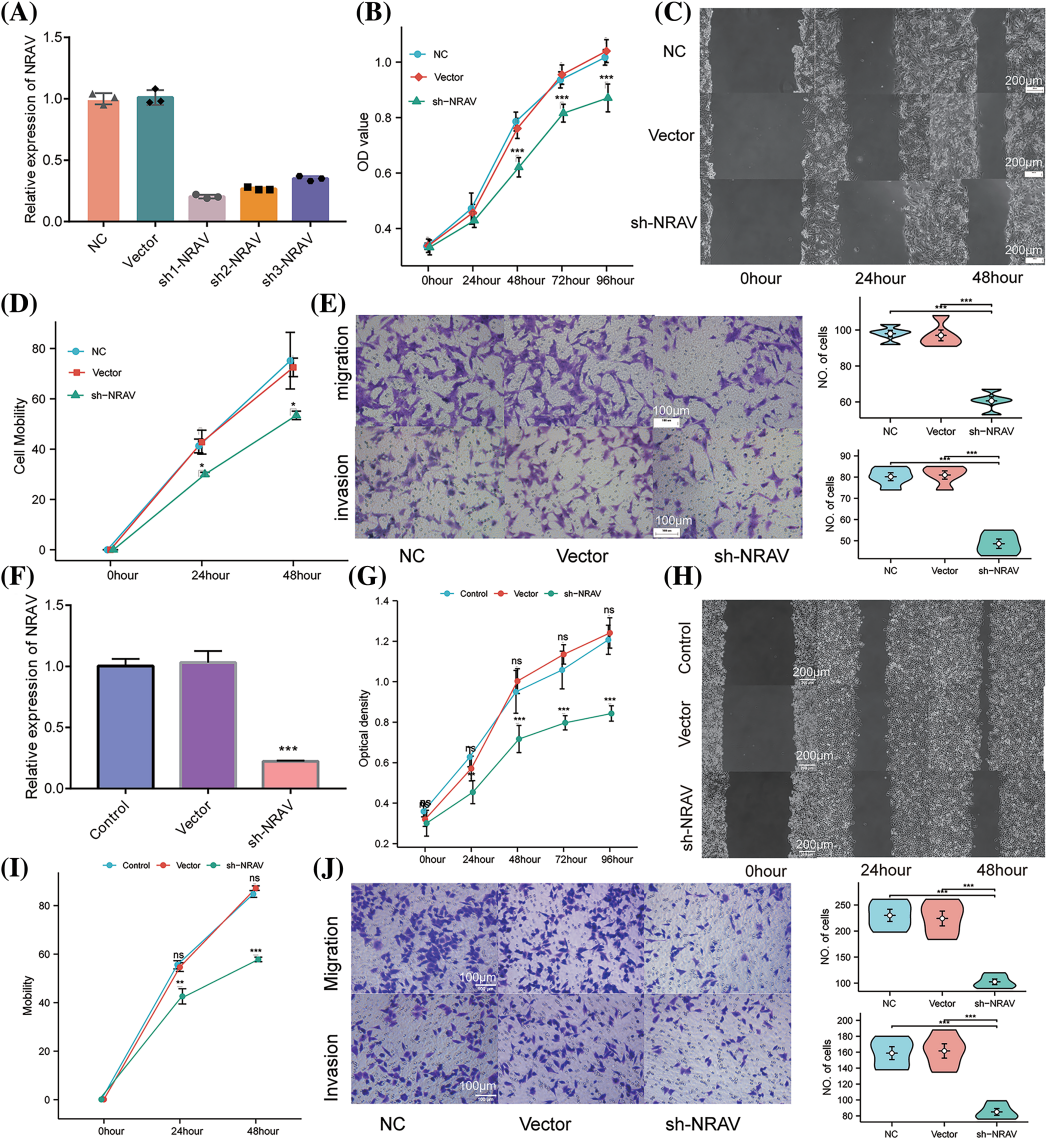
Knockdown of NRAV affects proliferation and migration of Huh7/HCCLM3 cells. (A) NRAV knockdown efficiency is measured through real-time PCR in Huh7 cells. (B) An assay using CCK8 detected Huh7 cell proliferation. (C and D) A cell scratch assay was used to determine the ability of the cells to migrate. (E) In order to measure cell migration and invasion, Tranwell analysis was conducted. (F) Tests of NRAV knockdown efficiency in HCCLM3 cells by real-time PCR. (G) Assays for CCK8 detected proliferation. (H and I) An assay for cell migration was performed using a scratch test. (J) Cell migration and invasion were performed by tranwell analysis. ns, *p* ≥ 0.05, *p < 0.05, **p < 0.01, ***p < 0.001.

Previous research has established the upregulation of NRAV in HCC, which was further strongly correlated with a decreased survival rate. To compare the knockdown efficiency of different shRNA sequences and to avoid off-target effects, we each transfected three different shRNA plasmids into HUH7 cells and performed RT-PCR. According to the RT-PCR results, the highest knockdown efficiency was achieved by sh1-NRAV in mRNA levels (Fig. 4A). Subsequently, the effect of NRAV on HCC cell proliferation was determined by examining cell proliferators using CCK8 assays. The shNRAV transfection group exhibited a significantly reduced proliferation ability at 48 h compared to that of the normal cell group and negative vector group (Fig. 4B). Furthermore, NRAV knockout cells exhibited significantly reduced healing abilities, as evidenced by the cell scratch test (Figs. 4C–4D). Furthermore, transwell chamber experiments demonstrated that NRAV knockout inhibited both migration ability and invasion ability (Fig. 4E). RT-PCR was then used to detect stable knockdown efficiency of NRAV in HCCLM3 (Fig. 4F). Additionally, the CCK8 assay showed that NRAV knockout significantly inhibited HCCLM3 cells proliferation at 48 h (Fig. 4G). Finally, wound healing assays, transwell migration assays and Matrigel invasion assays showed that the knockdown of NRAV markedly reduced the migration/invasion ability of HCCLM3 cells (Figs. 4H–4J).

### The role of NRAV in EMT, pyroptosis, immune response, and autophagy

**Figure 5:**
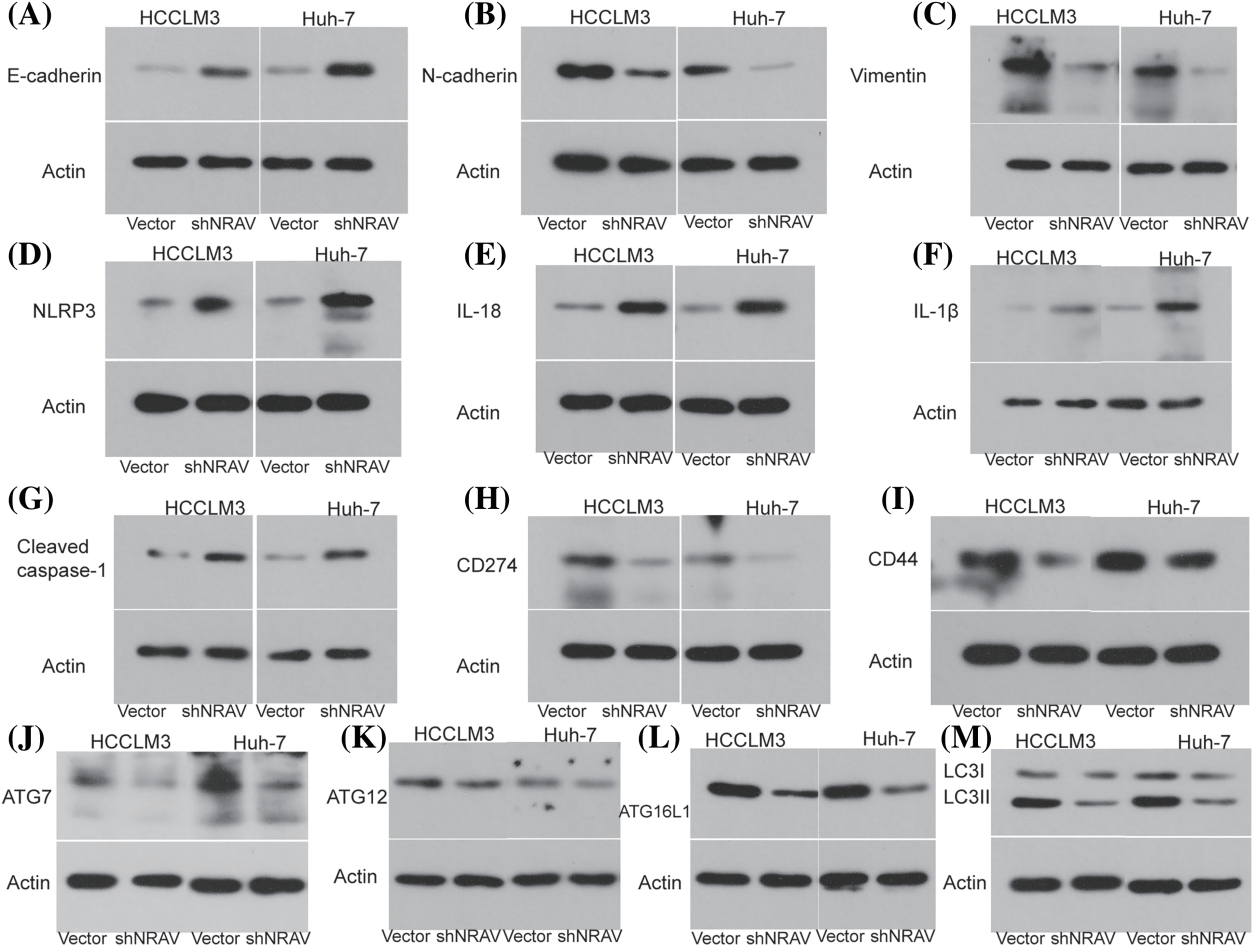
NRAV may be involved in regulating EMT, autophagy, pyroptosis, and immune. The protein levels of N-cadherin (A), E-cadherin (B), Vimentin (C), NLRP3 (D), IL-18 (E), IL-1β (F), Cleaved caspase-1 (G), CD274 (H), CD44 (I), ATG7 (J), ATG12 (K), ATG16L1 (L), and LC3 I/LC II (M) were detected by Weston blot after knockdown NRAV.

Previous studies demonstrated that NRAV was correlated with genes involved in EMT, pyroptosis, immune response, and autophagy, however, the role of the NRAV needs further elucidation. Here, we examined the expression of several key genes in HCCLM3 and Huh-7 cells following NRAV knockdown using Weston blot analysis. Subsequently, we conducted measurements on the levels of EMT-associated markers in HCC cells. Our findings revealed that the silencing of NRAV decreased N-cadherin and Vimentin expression, but increased the expression of E-cadherin (Figs. 5A–5C). We also saw increased pyroptosis-related markers (NLRP3, IL-18, IL-1β, and Cleaved caspase-1) and protein level upon NRAV knockdown (Figs. 5D–5G). In addition, we found that CD274 and CD44 were significantly downregulated after NRAV knockdown (Figs. 5H and 5I). Moreover, Knockdown of NRAV causes down-regulation of autophagy-related genes (ATG7, ATG12, ATG16L1, LC3I/LCII) (Figs. 5J–5M).

## Discussion

A growing body of evidence indicating the significant involvement of LncRNAs in the initiation and progression of cancer. NRAV has been shown to inhibit the transcription of multiple interferon-stimulated genes and is thought to be involved in interferon-mediated antiviral response [13]. Furthermore, mounting clinical evidence indicates that NRAV serves as an independent risk factor for various cancers [14–16]. NRAV was also identified as an immune-related and ferroptosis-related lncRNA in HCC [17,18]. Nevertheless, the expression of NRAV across different types of cancer remains poorly characterized. Considering this knowledge gap, our research endeavors to comprehensively examine the expression patterns of NRAV in various cancer types, analyze its association with clinicopathological characteristics in patients, and evaluate its biological functions in HCC through both *in vitro* and *in vivo* models.

In this study, NRAV expression was abnormally high in different types of tumors, but low in KICH tumor tissues based on the TCGA database. Kaplan-Meier survival analysis indicates that high levels of NRAV are associated with poor overall survival (OS) rates in patients with LGG, LIHC, LUAD, LUSC, PAAD, PRAD, MESO, and THCA. The development of cancer is increasingly associated with abnormal interactions between tumor cells and their microenvironment [19–21]. The tumor immune microenvironment is the main battleground where cancer cells interact with immune cells [22,23]. Recent research has suggested that NRAV plays a role in showing cancer progression and maintaining genomic stability. Our study demonstrates a positive association between NRAV and various immune cell types, including B cells, CD4+ T cells, CD8+ T cells, neutrophils, macrophages, and DC cells, in multiple cancer types such as KIRC, LGG, PRAD, LIHC, USC, GBMLGG, KICH, and DLBC. In addition, our ssGSEA analysis found a substantial positive association between NRAV and immune cells invading Th2 cells, TFH, macrophages, NK CD56bright cells, and T helper cells in LIHC, all of which could be leveraged for targeted treatment responses or prognostic information.

Studies have indicated that activated Th2 cells secrete the IL-4 cytokine to suppress tumor rejection and promote tumor growth [24]. Macrophage M2 inhibits T cell proliferation and differentiation and promotes tumor cell proliferation and angiogenesis [25]. Furthermore, CD56 homodimers function as adhesion molecules among cancer cells and as communication and organization agents within the tumor microenvironment, these heterodimers have been associated with advanced stages or poor prognosis in several cancers [26–28]. Together, our results suggest that activated Th2 cells produce more IL-4, promote macrophage M2 polarization, and increase CD56 homodimers, thereby advancing tumor progression, especially in LIHC. Cancer can co-opt immune checkpoint pathways, which normally prevent collateral damage from anti-microbial immune responses, to achieve immune evasion [29]. Increasing evidence indicates that epithelial-mesenchymal cell transformation, autophagy, and pyroptosis can contribute to cancer progression, metastasis, and drug resistance [30–32]. Here, we conducted GO and KEGG enrichment analyses, which revealed that the co-expression molecules of NRAV are predominantly associated with RNA transport, cell cycle, and ribosome biogenesis in eukaryotes. These pathways play a significant role in tumor development, as cancer cells typically have disrupted cell cycle checkpoints, and increasing ribosome biogenesis is necessary to facilitate cancer cell growth and proliferation [33,34]. Furthermore, we carried out a clinical prognosis study evaluating the abnormal expression of NRAV in HCC, including survival status, pathologic T stage, histologic grade, and vascular invasion. Consistent with our expectations, our findings demonstrated a significant reduction in proliferation, migration, and invasion capabilities of HCCLM3 and Huh7 cells upon NRAV knockdown. Our findings suggest that NRAV is a promising biomarker that plays an essential role in the tumor progression of patients with HCC,therefore, NRAV could be a foundation for the development of targeted therapeutic strategies.

However, certain limitations need to be acknowledged in the current study. While we observed a correlation between NRAV and genes associated with EMT, pyroptosis, immune response, and autophagy, suggesting their potential significance in the pathogenesis and progression of HCC as well as their suitability as therapeutic targets, further clinical investigations are needed to ascertain their precise roles and underlying mechanisms. Secondly, this study validated NRAV function only *in vitro* cell, further *in vivo* validation could strengthen the experimental design and provide more robust evidence for the role of NRAV in HCC. Additionally, this study exclusively concentrated on the examination of gene expression data, neglecting the investigation of alternative variables that could potentially influence the onset of HCC, such as environmental exposures or lifestyle factors.

## Conclusions

In conclusion, our study provides significant new insights into the role of NRAV in cancer development and progression. We shed light on the potential mechanisms underlying NRAV’s involvement in cancer pathogenesis and validated the expression and function of NRAV in HCC cells both *in vitro* and *in vivo*. Furthermore, our study has several strengths, including its use of large-scale data analysis and biological validation experiments to investigate the role of NRAV in cancer. Overall, our findings reveal the complex mechanisms by which NRAV contributes to cancer pathogenesis and progression. These results serve as a solid foundation for future research endeavors to develop innovative approaches for the prevention and treatment of HCC.

## Supplementary Materials

Figure S1 **Correlation analysis.** (A) Correlation analysis between NRAV and immune cell infiltration in pan-cancer. (B) Correlation analysis between the expression of NRAV and immune checkpoint inhibitors. (C) Coexpression analysis of NRAV expression and autophagic-related molecules. *p<0.05; **p<0.01; ***p<0.001, ****p<0.0001.

Figure S2 **Correlation analysis.** (A) Correlation analysis of NRAV expression and epithelial-mesenchymal transition-related molecules. (B) Correlation analysis of NRAV expression and pyroptosis-related molecules. (C) Correlation analysis of NRAV expression and Tumor mutation burden. (D) Correlation analysis of NRAV expression and Microsatellite instability. *p<0.05, **p<0.01, ***p<0.001.

Figure S3 **Functional enrichment analysis of NRAV.** (A) The ssGSEA algorithm was employed to evaluate the abundance of immune cell infiltration in HCC. (B) Top 20 co-expression molecules interacted with NRAV (C, D). Construction of GO and KEGG interaction networks. *p<0.05, **p<0.01.

Figure S4 Single-cell expression of NRAV in hepatocellular carcinoma. (A) The distribution of cell types in LIHC GSE166635 cohort. (B, C) The single cell expression of NRAV in GSE166635 cohort.

## Data Availability

The data used to support the findings of this study is included within the article, and the data are available from the corresponding author upon request.

## Supplementary Materials

The supplementary material is available online at https://doi.org/10.32604/or.2023.043575.
