# Supplementary figures and images for: Investigation of the feasibility of NRAV as a biomarker for hepatocellular carcinoma
Source: Oncol Res. 2024 Mar 20;32(4):717–26. doi: 10.32604/or.2023.043575 (PMC10972727; doi:10.32604/or.2023.043575)

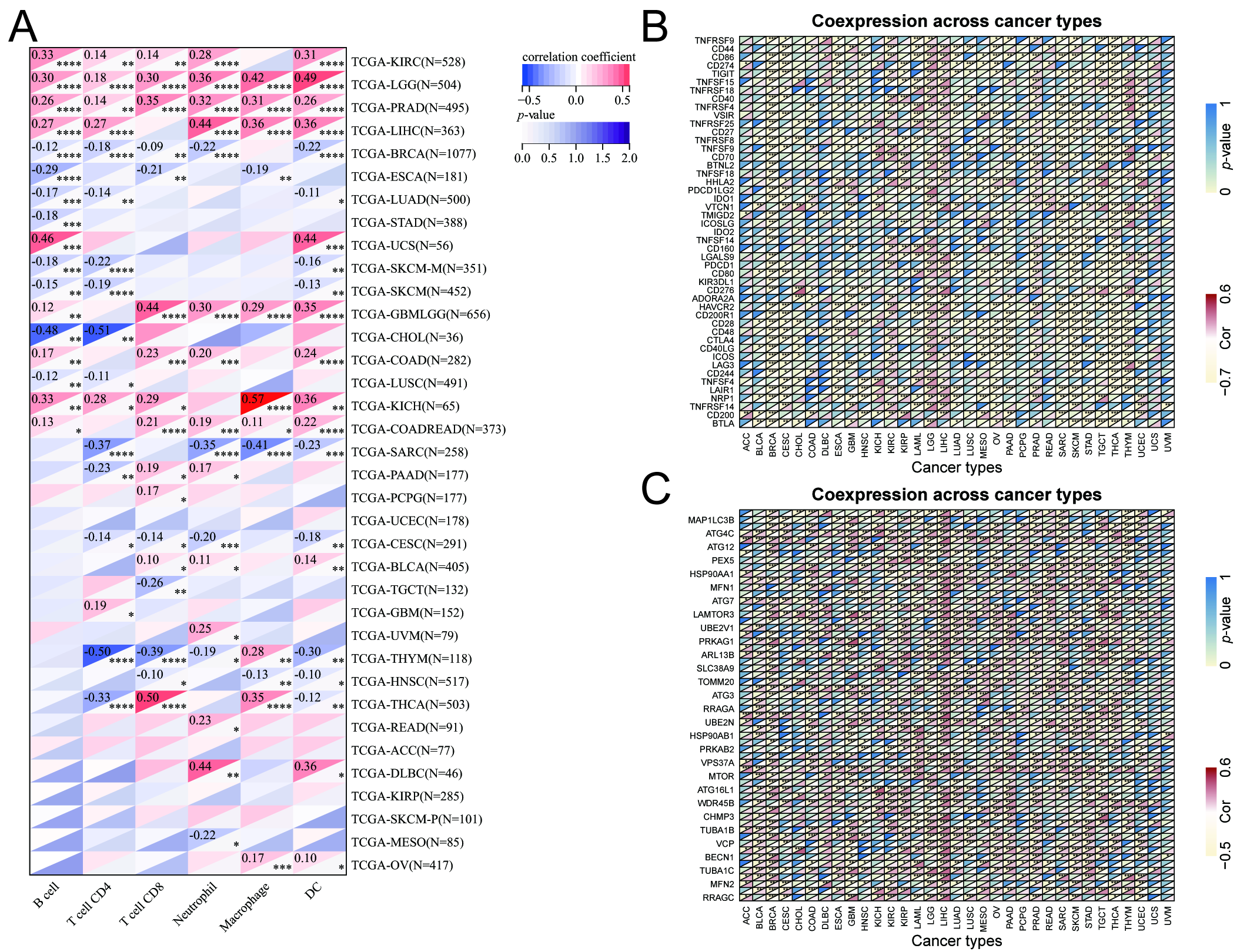

Supplement: Figure S1 [file OncolRes-32-43575-s001.tif]

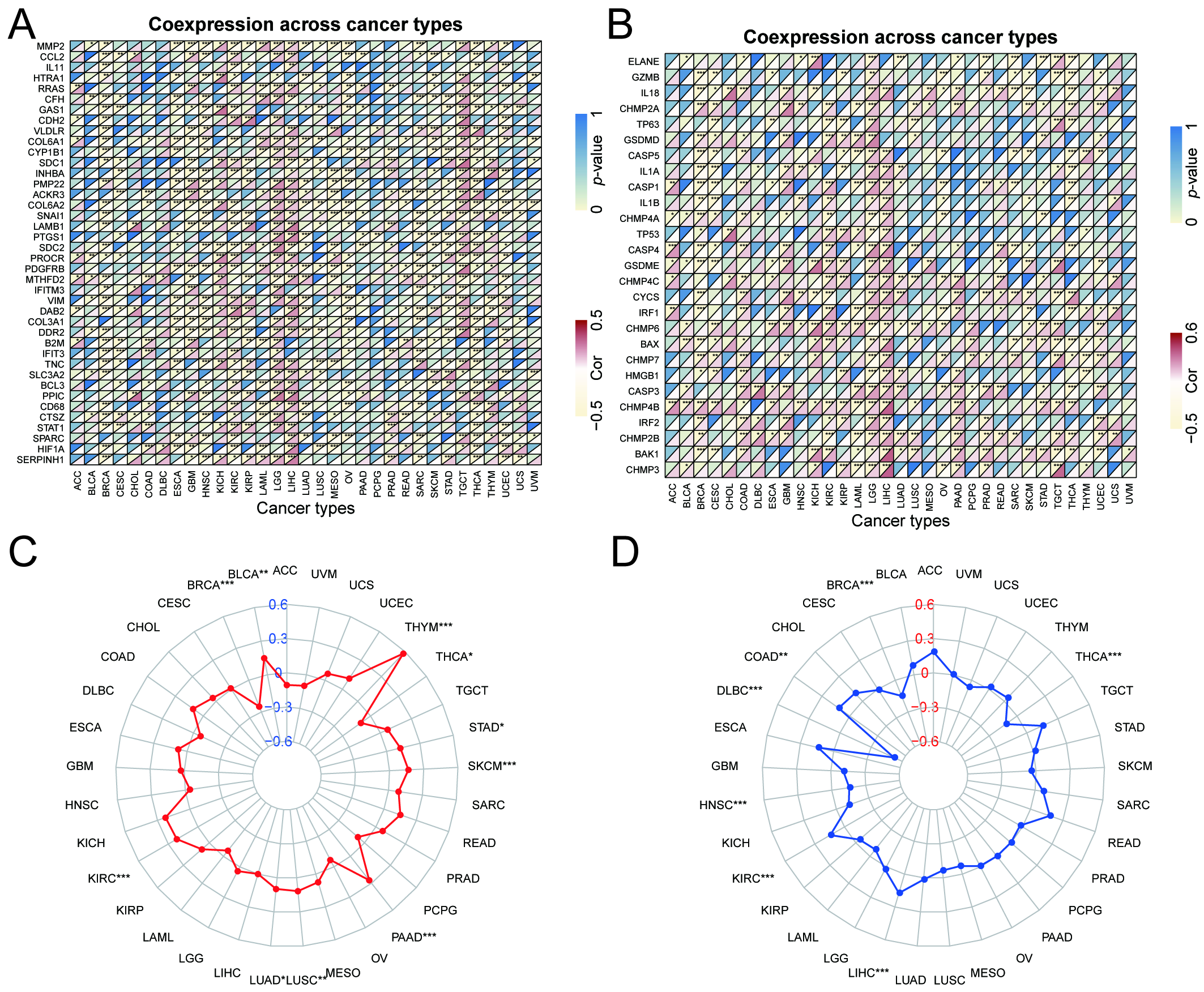

Supplement: Figure S2 [file OncolRes-32-43575-s002.tif]

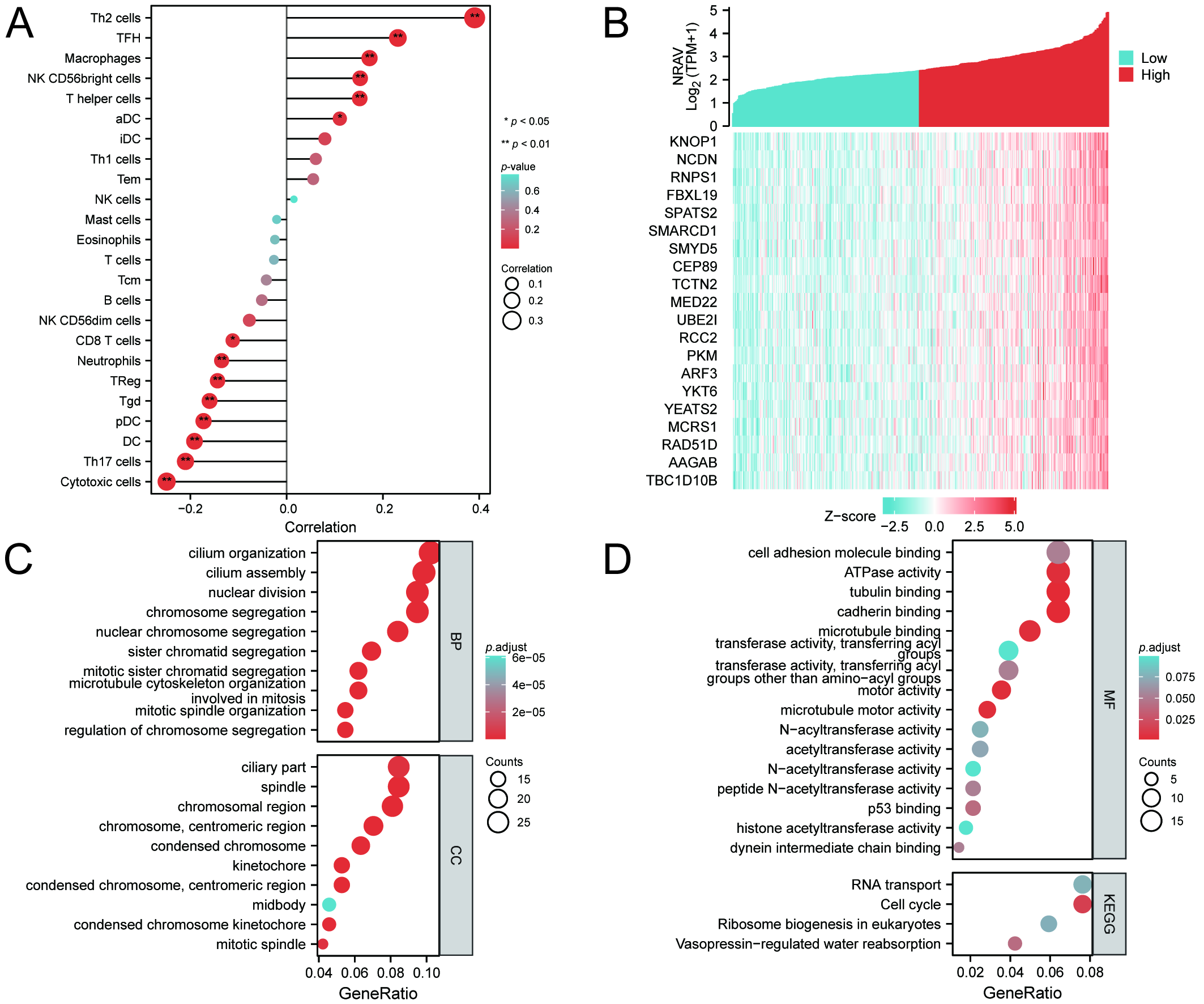

Supplement: Figure S3 [file OncolRes-32-43575-s003.tif]

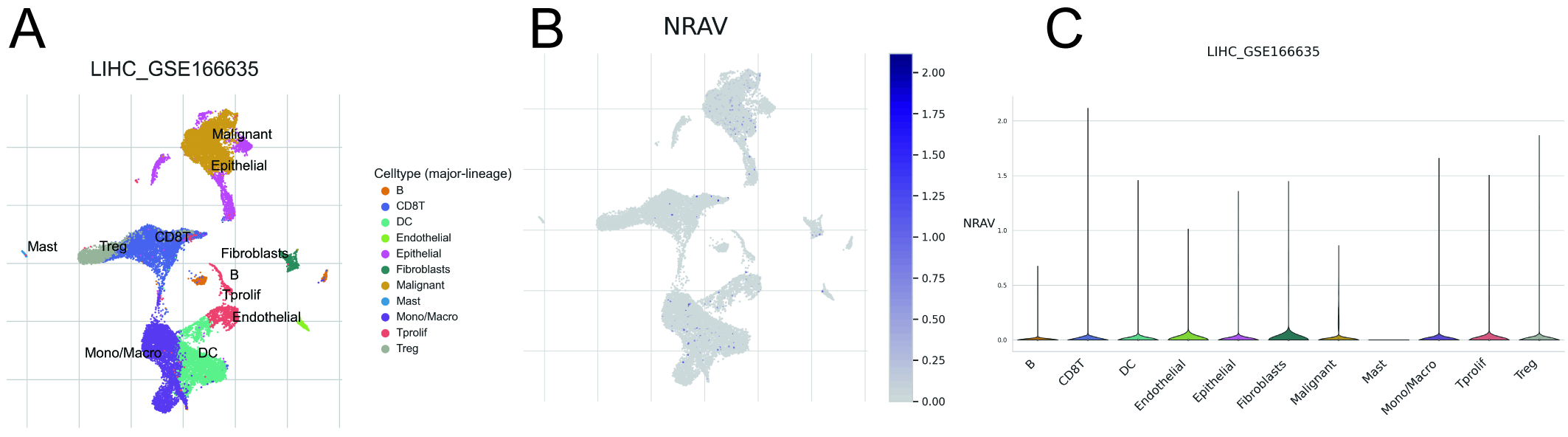

Supplement: Figure S4 [file OncolRes-32-43575-s004.tif]
